# Carotid Stenting in Carotid Stenosis Management—A Gamechanger After CREST-2? A Neuroradiological Perspective on a Potential Paradigm Shift

**DOI:** 10.1007/s00062-025-01602-8

**Published:** 2026-01-14

**Authors:** Olav Jansen, Fritz Wodarg

**Affiliations:** Department of Radiology and Neuroradiology, Universitätsklinikum Schleswig-Holstein – Campus Kiel, Kiel, Germany

The recently published CREST-2 trial [1] represents a potentially pivotal moment in the management of asymptomatic extracranial carotid artery stenosis. The accompanying *NEJM* editorial by Brown and Bonati [2] appropriately highlights the effectiveness of optimized medical therapy. However, a focused interpretation from the perspective of neuroradiology and neurointervention is essential and leads to a partially different conclusion. Rather than suggesting that asymptomatic stenoses should be managed conservatively in general, the results indicate that intervention—particularly carotid artery stenting (CAS)—should be applied when treatment is necessary, and under optimal conditions, can be considered a leading option.

CREST‑2 compared interventional therapy (either CAS or carotid endarterectomy [CEA] combined with medical treatment) versus medical therapy alone. While the conservative arm reported low event rates, the intervention arms also demonstrated very low periprocedural complication rates—significantly lower than in earlier generations of trials. This finding becomes especially relevant when contextualized with data from ACST‑2 [3], where CAS showed near equivalence to CEA in asymptomatic patients.

Looking retrospectively at landmark studies in symptomatic stenosis such as SPACE‑1, CREST [4], EVA-3S [5] and ICSS [6], a clear trend becomes evident: Over the last decade, CAS outcomes have significantly improved. Current data suggest that CAS is now highly competitive with, and in selected patients potentially superior to, CEA—provided that the procedure is performed by experienced operators in high-volume neurointerventional centres.

Several factors drive this progress:

- advanced stent and embolic protection technologies,
- improved catheter performance,
- and significantly greater interventional expertise, strengthened by the rise of endovascular stroke therapy.

From a neurointerventional viewpoint, CAS should increasingly be regarded as a preferred option, especially for patients with high-grade asymptomatic stenosis.

## Implications for Future Guidelines

Given the accumulating high-quality data, existing guideline recommendations should be revisited. Earlier guideline versions often favoured CEA based on historical evidence, whereas current evidence supports a more balanced perspective, with CAS now established as a competitive first-line therapy in suitably selected patients. This is increasingly reflected in recent neurovascular reviews, including [8], who emphasise that advances in stent technology and procedural safety justify broader use of CAS, especially when performed by appropriately trained specialists.

## Future Direction: Precision Identification of High-risk Patients

Improved medical therapy has effectively reduced event rates. However, risk remains elevated in patients with very high-grade stenosis or vulnerable plaque morphology or in patients with progressive stenosis. Rather than a general shift towards conservative management, a risk-based, precision medicine approach is required.

Modern neuroradiology plays a central role in this transition, with:

- advanced plaque imaging (CT, MRI),
- quantitative techniques and radiomics,
- and integration of haemodynamic parameters.

Future trials should focus on identifying high-risk profiles through imaging-based risk stratification. In such patients, CAS should be considered the treatment of choice.

## Conclusion

The evolving evidence supports moving from the traditional question *“stenting or not?”* towards the clinically more relevant consideration:

“When and for whom is CAS the optimal treatment strategy?”

With technological advances and growing neurointerventional expertise, carotid artery stenting has transitioned from an alternative option to a leading interventional strategy in carefully selected patients. CREST‑2 and related evidence herald a paradigm shift that must be reflected in upcoming guidelines and clinical decision-making.
